# Identifying feasible metabolic routes in *Mycobacterium smegmatis* and possible alterations under diverse nutrient conditions

**DOI:** 10.1186/s12866-014-0276-5

**Published:** 2014-11-18

**Authors:** Priyanka Baloni, Jyothi Padiadpu, Anupam Singh, Kuldeepkumar R Gupta, Nagasuma Chandra

**Affiliations:** Molecular Biophysics Unit, IISc, Bangalore, 560012 India; Supercomputer Education and Research Centre, IISc, Bangalore, 560012 India; Department of Biochemistry, IISc, Bangalore, 560012 India

**Keywords:** *Mycobacterium smegmatis*, Feasible metabolic paths, Phenotypic microarray, Gene expression, Gene redundancy, Adaptation

## Abstract

**Background:**

Many studies on *M. tuberculosis* have emerged from using *M. smegmatis* MC^*2*^155 (Msm), since they share significant similarities and yet Msm is non-pathogenic and faster growing. Although several individual molecules have been studied from Msm, many questions remain open about its metabolism as a whole and its capability to be versatile. Adaptability and versatility are emergent properties of a system, warranting a molecular systems perspective to understand them.

**Results:**

We identify feasible metabolic pathways in Msm in reference condition with transcriptome, phenotypic microarray, along with functional annotation of the genome. Together with transcriptome data, specific genes from a set of alternatives have been mapped onto different pathways. About 257 metabolic pathways can be considered to be feasible in Msm. Next, we probe cellular metabolism with an array of alternative carbon and nitrogen sources and identify those that are utilized and favour growth as well as those that do not support growth. In all, about 135 points in the entire metabolic map are probed. Analyzing growth patterns under these conditions, lead us to hypothesize different pathways that can become active in various conditions and possible alternate routes that may be induced, thus explaining the observed physiological adaptations.

**Conclusions:**

The study provides the first detailed analysis of feasible pathways towards adaptability. We obtain mechanistic insights that explain observed phenotypic behaviour by studying gene-expression profiles and pathways inferred from the genome sequence. Comparison of transcriptome and phenome analysis of Msm and Mtb provides a rationale for understanding commonalities in metabolic adaptability.

**Electronic supplementary material:**

The online version of this article (doi:10.1186/s12866-014-0276-5) contains supplementary material, which is available to authorized users.

## Background

*Mycobacterium smegmatis* MC^2^ 155 has been extensively used as a model organism to study various processes in *Mycobacterium tuberculosis* (Mtb). It closely resembles Mtb, and at the same time is non-pathogenic and has the added advantage of a much shorter doubling time than Mtb, making it both safe and practical to culture in the laboratory [1,2]. Resemblances between the two are seen at various levels. The two species show similar reactions to acid-fast staining, have similar cell wall structures, both synthesize mycothiol, exhibit adaptation in microaerobic conditions in absence of carbon, nitrogen and phosphorous and are capable of biofilm formation [3,4]. High levels of similarities are also seen in the individual genes between the two species [5,6]. Studies have been carried out in Msm to screen for probable drug candidates for tuberculosis [7-9].

Despite the use of Msm for several decades now, very little is understood about it from a molecular systems perspective, principally because majority of studies have focused on individual molecules. Although, the genome of Msm has been sequenced, there are no published articles reporting comprehensive analysis and annotation [10]. It is also known that the genome has high extent of redundancy. From conventional microbiology studies, it has been well known that the bacteria can grow under a variety of nutrient conditions including several different carbon and nitrogen sources [11,12]. Msm is also known to occur in many environmental niches [11]. There is however, no clear understanding about how the bacterium is able to exhibit such versatility. Adaptability is essentially systems property and cannot be explicitly explained by studying molecules individually [13]. Hence a systems approach is necessary to understand it [14].

Whole genome sequences of hundreds of bacterial species are available, providing an excellent starting point for systems level analysis [15]. The ease of transcriptomics has led to higher-level data for many species in terms of genome-wide gene expression values, facilitating more realistic reconstruction of systems. However, to understand physical behavior of the organism, phenotypic data becomes essential [16]. Phenotypic microarray experiments, where growth patterns of a given system are studied under hundreds of conditions, provide a platform to record the phenotypic behaviour of the organism in a high-throughput manner. Indeed phenotypic microarray data has now been reported for several species [17,18]. At present, data from each of these studies are analysed independently and inferences made based on that. In principle, data from multiple perspectives of the same system although may seem disparate at the outset, should in principle be consistent and be able to provide cross-explanations for various observations. However, connecting diverse pieces of data is a daunting task, due to difficulty in obtaining genome-to-phenome mapping. The scale in terms of number of components required to be considered for genome-wide studies increases the complexity further. There are very few studies so far in literatures that report such an integrated view of an organism [19,20]. In this study, we obtain phenotypic data for Msm in 284 conditions, obtain transcriptome profiles for the reference condition and analyse the genome sequence for functional annotation and to identify alternate enzymes. We then integrate them together to identify feasible metabolic pathways in Msm in the reference condition and rationalize phenotypic behavior of Msm under different conditions.

## Results

### Description of the Msm genome

**Figure 1:**
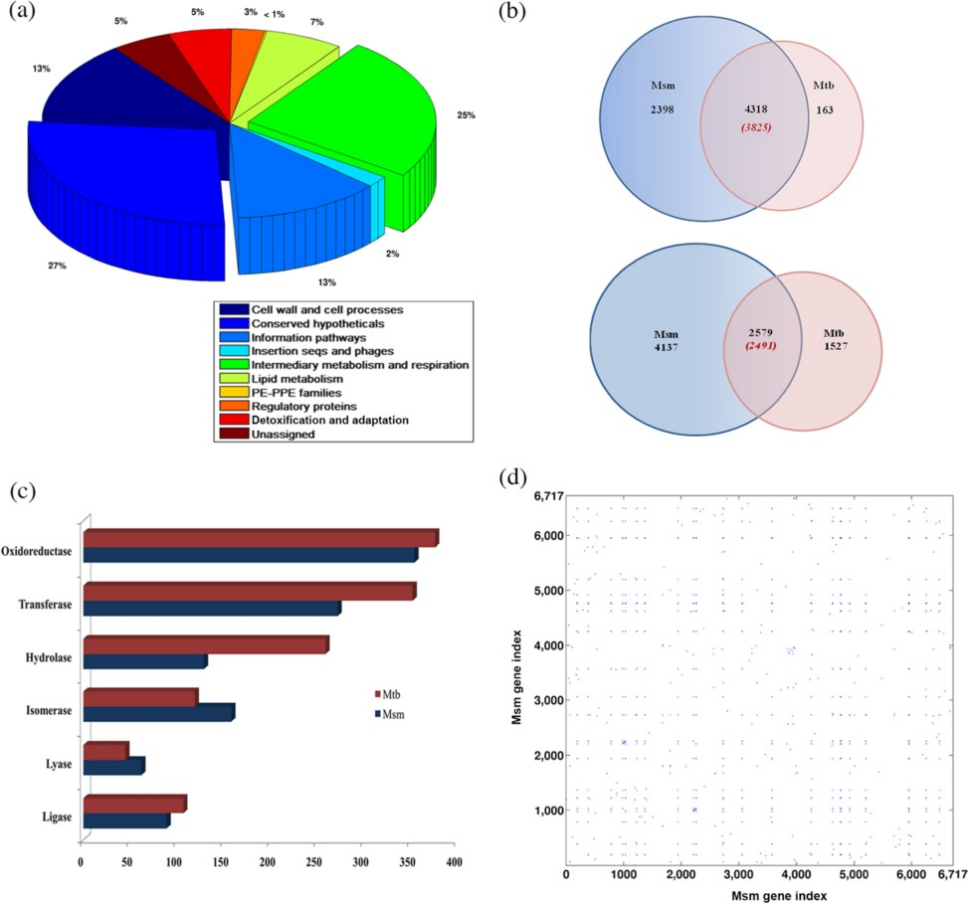
**Functional categories of** ***M.smegmatis*** **MC** ^**2**^ **155 genome and gene redundancy. (a)** The pie chart depicts distribution of the functional categories of Msm protein coding genes. **(b)** Venn diagrams depicting common and unique proteins present in *M. smegmatis* MC^2^ 155 and *M. tuberculosis* H37Rv based on sequence homology studies (the number mentioned in parentheses are the Mtb proteins common with Msm) (venn diagrams: top with 30% sequence similarity as threshold, bottom with 50% sequence similarity as threshold) **(c)** The distribution of Msm and Mtb enzymes classified according to their EC classes **(d)** Self similarity dot plot of the Msm genome depicting gene redundancy. X and y-axis represent gene indices.

*Mycobacterium smegmatis*, a non-pathogenic, saprophytic, acid-fast, rod-shaped bacterium, has a GC rich genome of 7 Mbp, consisting of about 6938 genes. MC^2^ 155, a reference strain of *M. smegmatis* is studied here, since it is widely used for experimental procedures because of its transformable morphotype [10,21-23]. Although genome sequence of *M. smegmatis* MC^2^ 155 (Msm) has been available, its genome annotation remains highly incomplete [10]. However, much can be gained by carrying out sequence analysis of Msm proteins and inferring function from well-annotated homologues in sequence databases, such as Mtb. Msm genome codes for 6716 distinct proteins of which 1064 are cellular enzymes. Homologues with either high confidence or previously assigned function in the sequence databases were identified for 6371 proteins, enabling transfer of Tuberculist functional categories [24] to the Msm proteins. No homologues were identified for about 345 genes and hence their function remained unassigned. Figure 1a illustrates distribution of functional categories assigned for the Msm genome. A detailed gene locus list and the assigned functional categories for Msm proteins are listed in the Additional file 1. The Venn diagram in Figure 1b depicts common and unique genes between Msm and Mtb, which indicate that majority of the Mtb proteins have homologues in Msm, leaving out only 343 proteins to be unique to Mtb. A large number of proteins, which sum up to nearly 2400, majorly being classified into conserved hypotheticals, are seen to be present in Msm but not in Mtb. Other features that stand out when Msm is compared to Mtb are (a) about 10 PE and PPE genes present in Msm, as compared to about 168 proteins in Mtb, (b) a larger proportion of genes, summing up to about 1800, belong to conserved hypotheticals and (c) a significant reduction of genes in the virulence category. About 1064 enzymes are identified in Msm as compared to about 1258 in Mtb. It can be seen in Figure 1c that the distribution across EC classes are similar in Mtb and Msm. There appears to be a marginally higher number of isomerases (158) and lyases (62) in Msm as compared to 119 and 45 in Mtb. The significance of this, if any, is not readily comprehendible. However, it has been suggested by Titgemeyer *et al.*, that Msm is a saprophyte unlike Mtb and may have evolved more isomerases to be able to utilize a wide range of carbon sources [11]. In any case, the height of the Msm genome is larger with an additional 2000 genes and an increase in the some categories can be easily expected. We in fact observe several instances of gene duplications. Given the difference in the genome sizes between Msm and Mtb, we systematically studied the extent of redundancy in the genome. Figure 1d indicates the extent of duplication in the genome, which includes about 170 proteins functionally identified as insertion sequences and transposases.

### Use of gene expression profiles to identify feasible metabolic pathways in Msm

#### The Msm transcriptome

**Figure 2:**
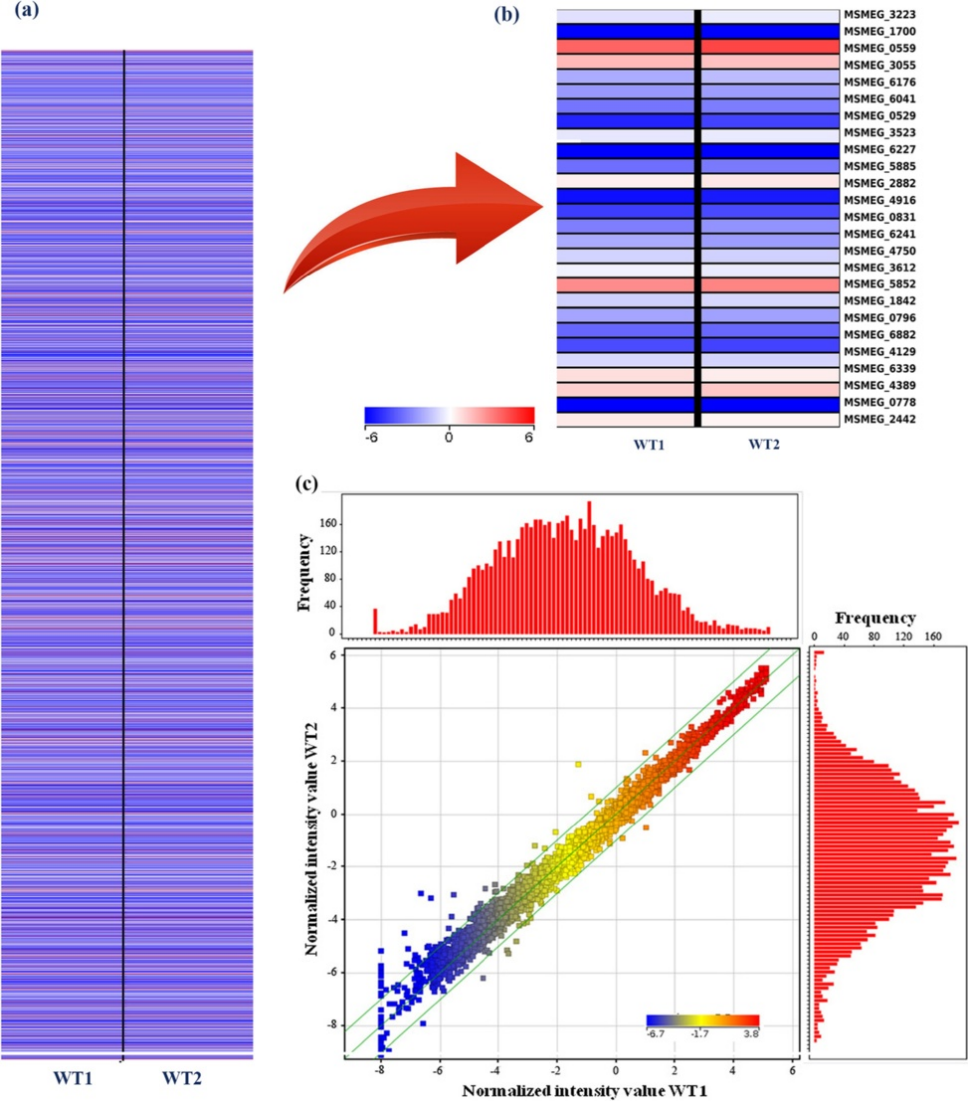
**Microarray data analysis and correlation between the biological replicates. (a)** Gene expression profile for all genes in Msm. **(b)** Enlarged portion of the heat map. The expression values are colour coded based upon the extent of expression where red indicates high levels of expression **(c)** Correlation plot for the biological replicates (R=0.99) and histograms depicting the distribution of the gene expression in the respective samples.

A gene-expression profile collected for the whole genome for cells grown in reference condition indicates that nearly the entire genome was probed in the array. The reference medium is composed of Middlebrook 7H9 broth, supplemented with glucose, glycerol and Tween 80 and reflects a standard wild type condition. We term this as the ‘reference condition’ hereafter. The expression patterns for the 2 biological replicates were seen to be highly similar (Figure 2) with a very high correlation coefficient (R = 0.99). Hence, average gene expression was calculated for the samples and the value has been used for other analyses [25]. Frequency distributions of normalized gene expression in the replicate arrays showing similar pattern in both, reflects a normal distribution. About 5018, 3278, 1597 and 676 genes out of 6761 genes probed using the microarray chip showed values higher than 25^th^, 50^th^, 75^th^ and 90^th^ percentile expression respectively (Additional file 2).

#### Identifying feasible metabolic pathways

**Figure 3:**
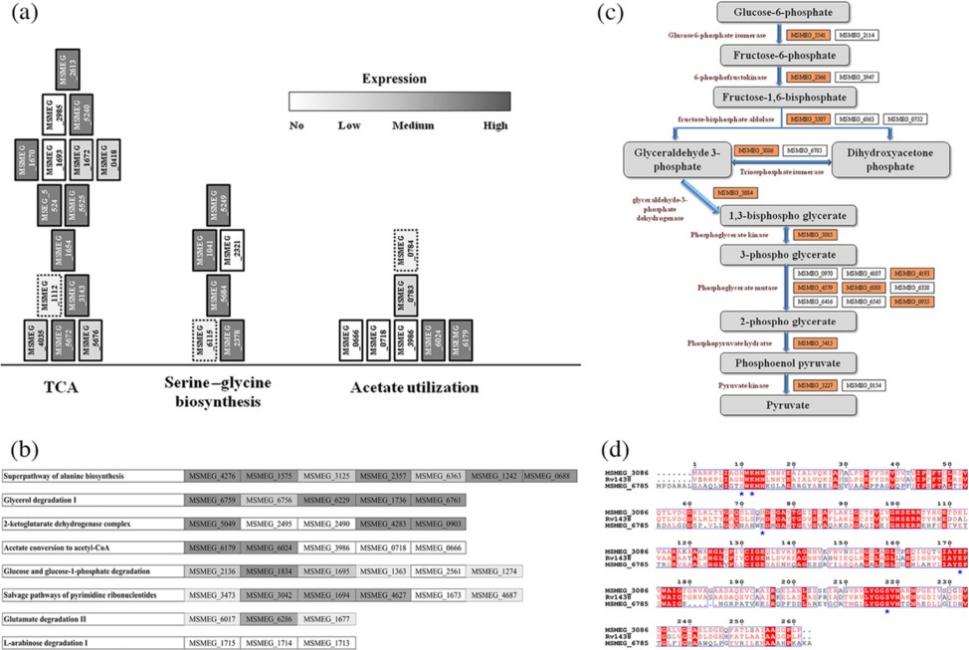
**Representation of the feasible pathways identified from transcriptomics and phenotypic analysis. (a)** Selected metabolic pathways with enzymes vertically ordered from bottom to top. They are colored in grey scale based on the extent of normalized gene expression. Multiple boxes present in a single layer depict the alternate genes encoding similar enzymes. **(b)** Representative pathways with the level of gene expression in reference condition **(c)** Schematic illustrating alternate enzymes being highly expressed in glycolysis, highlighted in orange **(d)** Multiple sequence alignment of tpi (triose phosphate isomerase) for Mtb and two Msm proteins. Sequence identity and conservation along with catalytic site residues (*) are shown.

In order to identify metabolic pathways active in Msm in log phase cultures in the reference condition, we map inferred enzyme abundances from gene expression values of individual genes, for all pathways in the organism listed in standard databases, KEGG and BioCyc [26,27]. For a pathway to be active, enzymes in it must be expressed in detectable quantities. Although gene-expression does not always directly correlate with protein abundances, transcription data is clearly suggestive of whether or not a protein is present in detectable quantities. Moderate correlation between expression levels and protein abundances has been reported for bacterial systems [25,28]. 338 pathways are identified for Msm that combines knowledge of experimentally known pathways from literature along with those inferred from genome sequence analysis. Genes corresponding to enzymes in expected pathways including central carbon metabolism, amino acid biosynthesis, purine and pyrimidine biosynthesis, fatty acid metabolism, mycolic acid biosynthesis are all expressed, as expected. Figure 3a and b shows gene expression pattern corresponding to enzymes in some pathways (data for all 338 pathways is given in Additional file 2), which illustrates that many pathways including those of central carbon metabolism, as expected, appear active owing to expression of all required genes. However expression levels vary from low to high, which is quite understandable owing to their individual biochemical properties. In all, 257 pathways can be considered to be active in the condition studied (for example, the first and second row in Figure 3b). There are about 14 pathways in which the genes show no expression (for example, last row in Figure 3b), and about 57 pathways where few genes in them are expressed whereas 75 pathways had most of the genes expressed. The latter have implications of ease of adaptability (discussed in a later section).

Another interesting feature that emerges from this analysis is the identification of the active enzyme(s) from the set of duplicates available for a given reaction. We analysed the expression patterns of 24 such sets of duplicate genes in terms of their contribution to their respective pathways. The trend that we observe indicates that in most cases only one of the possible alternatives is expressed (above median levels), while others are not, reflecting that there is minimisation of cellular expenditure in expressing redundant enzymes. In very few cases, more than one gene at a given step are simultaneously expressed. Figure 3c summarises our observations. Enzymes such as glucose-6-phosphate isomerase, 6-phosphofructokinase, fructose-bisphosphate aldolase, phosphoglycerate mutase, pyruvate kinase are encoded by more than 1 gene. MSMEG_3086, MSMEG_6785 code for triose phosphate isomerase enzyme. A multiple sequence alignment shown in Figure 3d indeed indicates that they are similar to each other [29]. It is interesting to observe that, of these two enzymes, only MSMEG_3086 is expressed. They are located 3674434 bp away from each other at positions in the chromosome. Similarly, other sets of paralogues are also located far apart from each other in the genome, indicating different transcriptional regulation. This analysis helps in associating specific genes to individual pathways, which becomes necessary for systems level modeling of metabolism, understanding of genomic deletions and any such genotype to phenotype associations. More examples of enzymes present in central carbon metabolism are shown in Additional file 3.

### Growth profiles of Msm observed using phenotypic microarray

**Figure 4:**
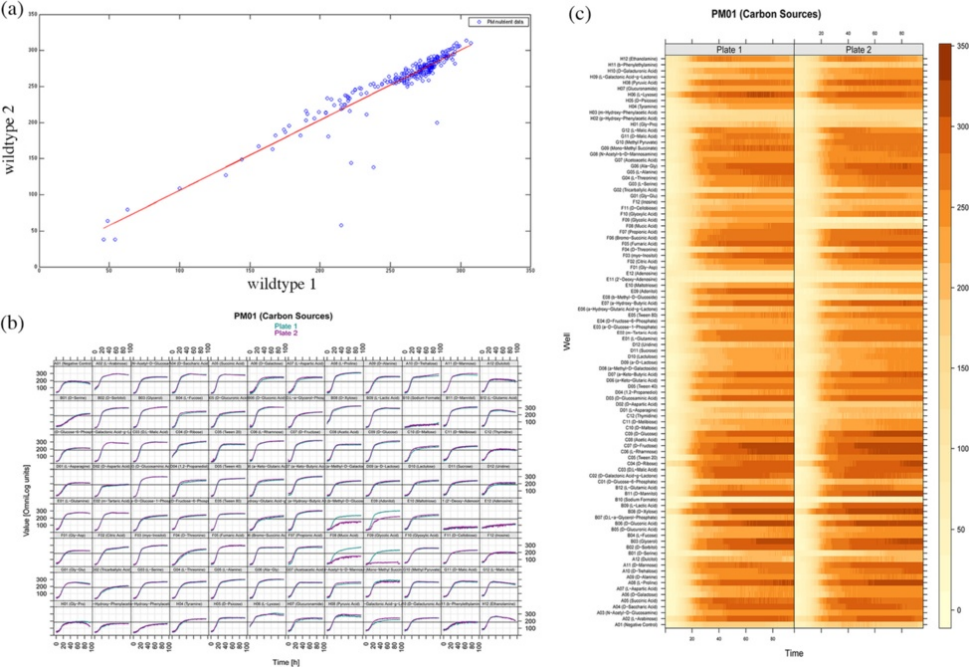
**Phenotypic microarray analysis. (a)** Correlation plot for nutrient plate (PM1, 3 and 5) of biological replicates at 48hrs. R value for the replicates was found to be 0.93. In scatterplot, Pearson correlation coefficient and linear regression line are shown **(b)** XY plot of carbon source plate (PM1) where x and y-axis represents time in hours and Omnilog units respectively. The Omnilog unit is a standard representation of respiration rate. **(c)** Level plot (PM1) indicating dye reduction which is coloured based on the Omnilog units. X-axis represents time in hours and y-axis represents nutrients present in the 96-well plate.

In order to characterize the growth profile of the organism under different nutrient conditions, phenotypic microarray (PM) analysis was carried out [30-33]. PM1, PM3 and PM5 plates were utilized for the experiment (plate compositions in Additional file 4). 284 different conditions were tested, of which 95 were carbon sources, 95 nitrogen sources and 94 were other nutrient supplements. As a validation exercise, batch culturing of Msm in the reference medium was carried out and the growth profile compared with that obtained from the well containing glucose in the PM1 plate. A consistent pattern in growth profiles was observed in the batch culture as well as the PM well, containing D-glucose as the carbon source (Additional file 5). We also observe high levels of consistency between the two biological replicates in PM plates. Scatter plot of kinetic data at 48 hours growth for all the nutrient sources shows high correlation (R = 0.93) between the biological duplicates (Figure 4a).

Figure 4b shows XY plots of the 95 conditions of PM1 plate illustrating growth curves under different carbon sources. We observe that certain carbon sources are more preferable for growth as compared to the others. Figure 4c showing level plot of PM1 plate capturing the extent of dye reduction and in turn the extent of respiration (XY and Level plot for PM3 and PM5 in Additional file 6). Correlation between the two replicates is evident from these plots as well. In the level plot, it can be seen that lyxose is a good carbon source, albeit poorer than glucose, whereas phenyl ethylamine is not. Pyruvate on the other hand is seen to support growth but only moderately. Few example of growth supporting compounds are summarized in Table 1. Similar insights are obtained for all the carbon and nitrogen sources studied here, summarized in Additional file 4.

**Table 1:** **List of substrates used as carbon source, respective metabolic pathways and genes coding for enzymes catalyzing the substrates**

**Source**	**Source type and metabolic pathways**	**Gene locus**	**Enzyme catalyzing substrate utilization**
L-Proline	Aminoacid, TCA, oxologlutarate, Glu	0943, 1413, 2681	pyrroline-5-carboxylate reductase, ornithine-oxo-acid transaminase, proline imino-peptidase
D-Xylose	Carbohydrate, Pentose and glucuronate interconversions	6021	xylose isomerase
D- Mannitol	Carbohydrate, Glycolysis, fructose, mannose	5576	D-mannonate oxidoreductase
L-Rhamnose	Carbohydrate, Glycolysis, fructose, mannose	0589	L-rhamnose isomerase
D- Fructose	Carbohydrate, Glycolysis, fructose	3094 , 0085	oxidoreductase, zinc-binding dehydrogenase, PTS system, Fru family protein, IIABC components
Alpha- D Glucose	Carbohydrate, Glycolysis	2136, 1363	Phosphogluco mutase, glucokinase
L-Arabinose	Carbohydrate, Glycolysis, G3P, Pentose and glucuronate interconversions	1675	L-arabinose isomerase
D-Saccharic acid	Carbohydrate, Glycolysis Ascorbate and aldarate	6117, 0455	Glucarate dehydratase, aldehyde dehydrogenase
Succinic acid	Carboxylate, TCA	5524 , 5525, 0417, 0418	succinyl-CoA synthetase subunit alpha and beta
D-Mannose	Carbohydrate, Glycolysis, fructose, mannose	1834	Phosphomanno mutase/phosphogluco mutase
D-Sorbitol	Carbohydrate, Glycolysis, mannose	3094, 3605	oxidoreductase, zinc-binding dehydrogenase, sorbitol dehydrogenase
Glycerol	Carbohydrate, Glycolysis, glycerate	6229	glycerol kinase
D-Gluconic Acid	Carbohydrate, Glycolysis	1274, 0453	gluconolactonase, shikimate kinase
L-Lactic acid	Carboxylate	2512	lactate 2-monooxygenase
D-Galactonic acid - gamma -Lactone	Carboxylate, Glycolysis, Glactose metabolism	6177	Galactonate dehydratase
D,L-Malic acid	Carboxylate, TCA, malinate	2551, 2910	Malate:quinoneoxido reductase, Fumarate hydratase class
D-Ribose	Carbohydrate, Pentose and glucuronate interconversions	4585	ribokinase
1,2-Propanediol	Alcohol, Glycolysis, pyruvate metabolism	0496, 0497, 1546, 1547	Propanediol dehydratase
Alpha-Keto-Butyric Acid	Carboxylate, Glycine, serine and threonine metabolism	3183, 3532	threonine dehydratase, serine/threonine dehydratase family protein
Myo-Insitol	Carboxylate, Galactose Metabolism	2762, 3116, 3210	inositol-1-monophosphatase
Fumaric acid	Carboxylate, TCA	2985, 5240, 0417	Fumarate hydratase class I, anaerobic, fumarate reductase iron-sulfur subunit
L-Alanine	Aminoacid, TCA, oxaloacetate, Pyruvate	0688	aminotransferase
Pyruvic acid	Carboxylate, Pyruvate metabolism	2471,4323, 4711, 4712	pyruvate dehydrogenase

Msm is observed to have higher growth in presence of the carbon sources mentioned in the table. The gene locus mentioned should be read as MSMEG_(number).

Of all nutrient conditions studied, 167 nutrients support growth, 96 carbon and nitrogen sources show moderate growth, while 21 sources do not support any significant growth (Additional file 4). Some notable observations are: (i) Tween is considered to be a source of fatty acids such as oleic acids. Tween 80 is known to significantly promote aerobic growth by improving O_2_ transfer, while only a small amount is known to be degraded and metabolized through the TCA cycle as part of the central metabolism for biomass synthesis [34]. It is utilized when given as a sole carbon source but not in combination with glucose. When supplied as a carbon source, Msm has a longer log phase in the growth curve, while as a nitrogen source it is used very efficiently which is not seen in other mycobacteria (Table 2). (ii) Serine is known to be converted to pyruvate in the presence of L-serine ammonia lyase. The enzyme is expected to be expressed only in the absence of glucose and the pathway becomes active in anaerobic conditions, similar to that observed in *E. coli* [35]. L-Serine can be used as a carbon source by Msm but not by other mycobacterial species [31]. (iii) Alanine is also deaminated to produce pyruvate, which is then converted to CO_2_ and acetyl-CoA. The reaction is known to be catalysed by alanine dehydrogenase, which is also present in Mtb [36,37]. (iv) Acetic acid mediated growth is also observed in Msm, indicating the presence of active gluconeogenesis pathways. (v) Acetamide did not favour growth and is consistent with earlier reports that it supports growth only in specially constructed inducible strains with conditional expression [38]. (vi) Formate is typically utilized by bacteria as a carbon source in the tetrahydrofolate biosynthesis, but in Msm it did not support growth, as the other required essential compounds in the central metabolism cannot be synthesized from this compound.

**Table 2:** **Comparison between** ***M. tuberculosis*** **and** ***M. smegmatis*** **for nutrient utilization**

**Carbon source**	**Mtb**	**Msm**	**Carbon source**	**Mtb**	**Msm**
2-oxoglutarate	✓	NA	Oxalomalate	✓	NA
Acetate	✓	✓	**Propanoate**	X	✓
Acetoacetic acid	✓	NA	Pyruvate	✓	✓
Adenosine	X	X	**D-serine**	✓	X
D-alanine	✓	✓	D-tagatose	X	NA
L-alanine	✓	✓	D-trehalose	✓	✓
L-asparagine	✓	✓	Tween 20	✓	✓
Butyrate	✓	✓	Tween 40	✓	✓
Caproic acid	✓	✓	Tween 80	✓	✓
Citrate	✓	✓			
D-fructose-6-phosphate	✓	✓	**Nitrogen source**		
D-glucose-6-phosphate	✓	✓	L-Alanine	✓	✓
D-glucose	✓	✓	**Allantoin**	X	✓
L-glutamate	✓	✓	L-Asparagine	✓	✓
L-glutamine	✓	✓	**L-Aspartic acid**	X	✓
Glycerol	✓	✓	L-Cysteine	✓	✓
Glycine	✓	NA	D-Galactosamine	✓	✓
L-lactate	✓	✓	**D-Glucosamine**	X	✓
**D-malate**	X	✓	L-Glutamic acid	✓	✓
L-malate	✓	✓	L-Glutamine	✓	✓
**D-mannose**	X	✓	L-Ornithine	✓	✓
Methyl-pyruvate	✓	✓	D-Serine	✓	✓
Mono methyl-succinate	✓	✓	L-Serine	✓	✓
**N-acetyl-glucosamine**	X	✓	**L-Threonine**	X	✓

The nutrient sources in **bold** indicate the differential utilization by Msm and Mtb. (✓=growth, X=no growth, NA=not available).

**Figure 5:**
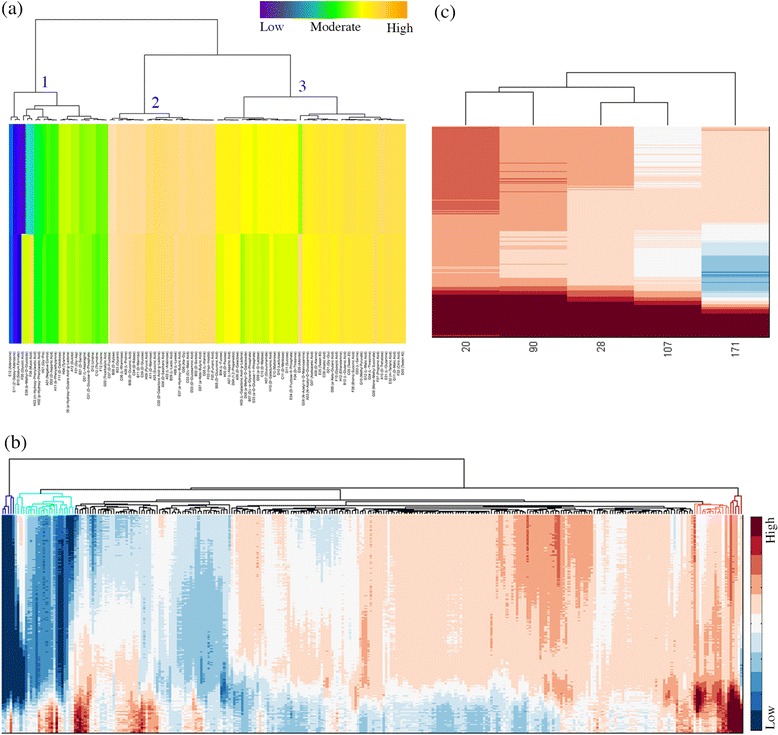
**Clustering analysis of PM plates. (a)** Clustering of different carbon sources. The colour bar indicates low to high utilization of different carbon sources. **(b)** Clustering of the nutrient mediated growth across all the plates, each column represents a well and each row represents time point (0-96 hrs, top to bottom), color bar ranges from blue to red depicting low to high respiration rate. **(c)** Cluster for high growth promoting nutrients. The well numbering and corresponding metabolite details for the three PM plates are given in the supplementary (Additional file 3).

From the XY and level plots, it can be seen that some conditions yield a similar phenotype. In order to identify which conditions show similar effect on the growth of the organism, a clustering exercise was carried out, from which distinct clusters were obtained depending upon the extent of utilization of the carbon source. The clustergram shown in Figure 5a indicates 3 major clusters as observed for PM1, the first referring to those conditions that do not support any significant growth, whereas clusters 2 and 3 refer to those showing high and moderate growth respectively. Carbon sources glucose, fructose, xylose, alanine, succinic acid and sorbitol all group into the high growth cluster while TCA intermediates, sucrose, maltose and Tween 20, Tween 40 and Tween 80 are found in the moderate growth category. Similarly for PM3 and PM5 plates, we find 2 major high growth and 4 moderate to lower growth clusters (clusters obtained for PM3 and PM5 are given in Additional file 7) referring to high growth and moderate growth categories. We also compare them across plates, by clustering them all together and find that the explored set of nutrient sources all map into six growth-pattern types (Figure 5b). Overall, nutrients enhancing growth of the organism were seen to be clustered together while those that do not support growth clustered separately. The nutrients such as hydroxylamine, 2-deoxy-adenosine, guanine and formic acid form a cluster together, all of them not capable of supporting growth in Msm. An enlarged portion of the figure is shown for the high growth cluster (Figure 5c), which describes the extent of variation in cellular respiration and thus growth under different conditions. For example, thymidine, phenylethylamine, inosine, mucic acid and alpha-methyl-D-glucoside group into one low growth cluster while D-galactose, L-aspartic acid, lactulose and L-fucose group into moderate growth cluster. It is interesting to observe that carbon sources D-xylose, L-lyxose and D-ribose group along with nitrogen sources uric acid and L-cysteine indicating that they have a similar influence on metabolism in the cell. These compounds enter metabolism at different points in the network and yet yield similar phenotypes perhaps due to a similar emergent effect.

### Rationalizing phenotypic behaviour by integrating transcriptome data with pathways

**Figure 6:**
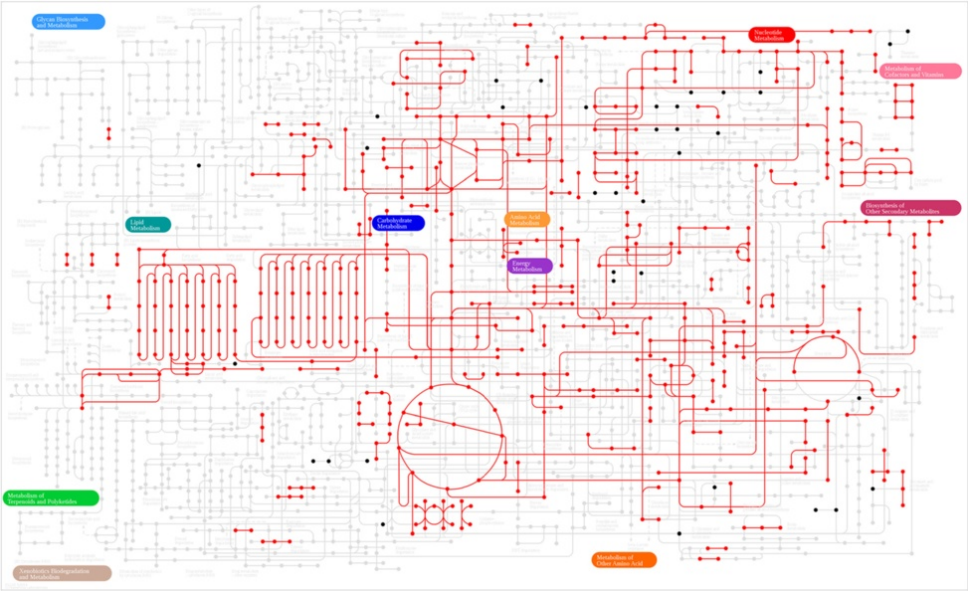
**Metabolic pathway map for Msm.** The KEGG metabolic map overlaid with gene expression data and nutrients utilized from PM analysis. The nodes represent metabolic compounds and edges represent genes involved in the reactions (red lines: gene expression, black dots: compounds identified from PM but not directly connected to enzymes expressed in reference condition).

Mapping gene expression values onto different enzymes in the metabolic network illustrate the various metabolic flows that are occurring in Msm in the reference condition, as shown in Figure 6. Pathways of the central carbon metabolism, TCA cycle, glyoxylate shunt, glycolysis and fatty acid biosynthesis, all appear to be feasible paths amongst the 73 super-pathways [27]. Among 284 conditions tested in PM, around 135 points mapped onto the KEGG metabolic network. Additional file 8 illustrates these points in a biochemical network diagram. Using this as a reference metabolic network, we attempt to rationalize observed phenotypic behaviour of Msm. The mapped compounds reflect that a vast portion of the network is indeed probed.

#### Transporters for uptake of nutrients

For a compound to serve as a nutrient source, it needs to be taken up by the cell. Such uptake takes place with the help of specific transporters. We identify transporters from the genome sequence and then feasibility of their activity through gene expression data. About 282 transporters were annotated by our analysis and amongst them we found about 60 to be expressed in the reference condition (glucose as carbon source) (shown in Additional file 9). It is known that a gene cluster comprising MSMEG_2116 to MSMEG_2120 forms a part of the glucose-sucrose subfamily in phosphotransferase system (PTS) [11]. The expression of this cluster seems to be lower but these are known to be constitutive as compared to other transporters. This PTS also comprises of trehalose, GlcNAc (N-acetylglucosamine), and dihydroxyacetone (MSMEG_2121 to MSMEG_2124) permeases which are expressed. The transporters for fructose such as MSMEG_6802, MSMEG_6803 and MSMEG_6804 seem to be expressed in the reference condition itself. Msm has glucose-6-phosphate isomerase (MSMEG_5541) for its utilization. Fructose is also known to have another mechanism of utilization via the expression of fructose-specific PTS composed of EI (ptsI), HPr (ptsH), and IIABCFru (fruA) (MSMEG_0084 to MSMEG_0088). However the expression here is lower except in the first locus. This cluster is known to be inducible in the presence of fructose as the sole carbon source. Indeed, high growth is observed in the PM plate, with fructose as the carbon source. Additional file 10 lists the possible transporters as deduced from the genome sequence and highlights those among them that are expressed under reference nutrition conditions. Transporters for glucose, xylose are seen to be expressed, providing first level explanation for utilization of these compounds as carbon or nitrogen sources. It has been reported that Msm can utilize different sugars indicating activation of various transporters and hence also changes in gene expression levels [11,39-41].

#### Connecting nutrient sources to metabolic pathways

Next, we study, if a given source compound can be mapped onto specific pathways in Msm, through which it can enter metabolism [18]. About 135 of these compounds are direct metabolites in the network and hence growth patterns with them are easily interpreted. Several more compounds can be linked with a metabolite in the network with one or few steps. In such cases, we study if the enzymes corresponding to their conversion can be detected in the genome. Additional file 4 lists these cases. One example is D-Mannitol, which is known to get converted into D-fructose then to fructose 6-phosphate, thus entering glycolysis [42]. A transporter for this can be traced from the genome sequence (MSMEG_5574). It is not expressed highly in the reference condition, but perhaps gets induced when mannitol is the sole carbon source. Similar behaviour is observed for trehalose, sorbitol and D-saccharic acid sources [11]. There are many lines of evidence from individual molecular biology studies to support the functional roles of these molecules [43-48]. Put together, they explain why these compounds serve as carbon or nitrogen sources that promote bacterial growth.

Another example is the conversion of serine into many central carbon metabolites through the glycolysis pathway and then to glycine and cysteine thus supporting growth [34]. The central carbon metabolism in Msm is represented in Additional file 3. Utilization of the range of carbon sources shows the repertoire of possibilities for metabolic pathways in the bacterium. Glycerol, arabinose, mannose, D-glucose and many other polyols, pentoses, hexose and also complex sugars enhance growth as supported by literature [39-41]. The alternate carbon sources such as L-proline, rhamnose, xylose and others are also being utilized for growth in Msm indicating these can induce their uptake and successful utilization. Glycerol can be taken up by a facilitator (MSMEG_6758) and used by the enzyme glycerol kinase (MSMEG_6759 shown to be expressed abundantly) to form glycerol 3-phosphate which can then enter central carbon metabolism. The observation about absorption and utilization of maltose is also in line with other experimental evidences, showing very low or retarded growth. Galactose and lactose show only moderate growth, consistent with the observation that the corresponding enzymes and transporters show poor expression values [11,39-41].

It is not clear whether there are any transporters for utilisation of trehalose from the external medium in Msm. Nevertheless, it seems to be enabling growth in the bacterium. It is possible that it can be involved in central carbon metabolism as well as be a component of the cell wall in the form of conjugates of mycolate, such as trehalose dimycolates and trehalose monomycolates [44,46]. Many of the TCA intermediates such as succinic acid, citric acid seem to promote growth. This is again consistent with the observation, that many of the central carbon metabolism genes are constitutively expressed in Msm. We also observe acetate and oleic acid (derived from Tween 80) being utilized for growth. This observation is consistent with known biochemical studies that glyoxylate shunt is prominent for anaplerosis in the bacterium allowing the utilization of acetate or fatty acids as the sole carbon sources while it allows the regeneration of the four-carbon malate from glyoxylate and acetyl-CoA for biosynthetic processes. The shunt can also replenish amino acids such as glycine and serine [34].

Amino acids such as L-Proline, L-Alanine and dipeptides such as L-Alanyl-Glycine seem to promote growth as nitrogen sources [37,49-51]. All other amino acids tested are also able to support growth either highly or moderately, indicating the ability of Msm to adapt to a wide variety of nitrogen sources and supplements. The genes involved in purine salvage pathways seem to be moderately expressed in Msm. Adenosine as a sole carbon source does not support growth, consistent with earlier suggestions in literature as well [31]. Examination of the gene expression values of enzymes involved in a pathway that salvages adenosine, indicates that the pathway is infeasible since enzymes adenosine deaminase, adenosine kinase and adenine phosphoribosyltransferase are virtually non-expressed under the conditions studied. However, when adenosine is supplied as a nitrogen source along with glucose as the carbon source, small extent of utilization is observed. An enzyme unique to mycobacteria, 5-methylthioadenosine phosphorylase (MSMEG_0990), that converts adenosine to adenine and alpha-D-ribose-phosphate is moderately expressed, perhaps presenting the only feasible way for adenosine utilization [52]. Thus the low activity of purine salvage pathways makes *de novo* biosynthesis of purine nucleotides highly essential for the survival of the organism, presenting targets for antimycobacterial drugs [53-56]. In fact, analog-based inhibitions of the de novo biosynthesis pathway enzymes are already under consideration as anti-tubercular drugs. Guanosine can be efficiently used by Msm as the same enzyme (MSMEG_0990) can cleave inosine and guanosine as well [52]. Overall, the differences in growth patterns under different conditions are explained by (a) presence or absence of a transporter for nutrient uptake, (b) presence and the expression level of the utilizing enzymes.

Microarray data of Mtb shows about one-fourth of the genes are consistently expressed under standard nutrition conditions in *in vitro* cultures [57]. Phenotypic microarray studies have been reported for Mtb, using a similar Biolog experimental setup [31,32]. Comparison of the growth patterns in Msm and Mtb, as observed from phenotypic microarray experiments, reveals that the two species show similar growth behaviour in most cases. This implies similar metabolic flow for most of the studied probes (Table 2). Exceptions to this are compounds D-malate, D-mannose, N-acetyl glucosamine, propoanoate, allantoin, L-aspartic acid and L-threonine which serve as nutrients to Msm but not to Mtb, while D-serine is the only compound that serves as a nutrient to Mtb but not to Msm (Table 2). Thus, overall, Msm can utilise most of the carbon sources and nutritional supplements as compared to Mtb and other mycobacterial species. Tween can be used as a sole carbon source in both but unlike in Mtb, it cannot be utilised in combination with glucose in Msm. Thus, it can be seen that 31 nutrient sources are common and 9 are unique between Msm and Mtb.

## Discussion

Phenotype of an organism is the cumulative effect of the genetic makeup and interaction of many composite molecules in the organism. Biochemical alterations in metabolism would be necessary to support [34,58] phenotypic variations of that organism. Given the high levels of interconnectedness in organisms, as evidenced by high complexity in genome-scale networks, there are many ways by which metabolic alterations can influence a system. Thus, it is important to evaluate the organism in a multitude of sets of scenarios that might occur in its environment. Phenotypic microarray studies offer such a platform where such evaluation of various different arrays of nutrient supplement and chemical environments can be carried out in a high throughput manner. Phenotypic microarrays have the added advantage of providing a direct readout of cellular respiration, enabling us to visualise and analyse growth patterns of the particular organism [13,14,19,31,33,59,60].

Most of the mycobacterial species exhibit common physiological traits such as adaptation to hypoxic conditions by maintaining itself in a dormant state. It is well known that Mtb survives inside the host by altering its metabolic requirements [61,62]. Msm has comparable physiological responses during dormancy as Mtb, thus making it a feasible model to study metabolic alterations and gain mechanistic insights [34,63].

Knowledge inferred from transcriptomic analysis, aids in unraveling the attainable metabolic routes in the organism. Adaptation to different environmental scenarios is due to induced variation in gene expression profile. However, it is a challenging task to predict phenotypic behaviour of the organism from its genotype. In order to rationalize the genome-phenome relationship, it has become essential to integrate information obtained from such high-throughput techniques. Integrating knowledge of phenotypic response in different conditions with the transcriptome data, as observed in this study, leads to a bird’s eye-view of genome-transcriptome–phenome pertaining to metabolism in mycobacteria. Such information can be used as direct inputs to build systems level models to comprehend large number of parameters simultaneously. The ultimate use of this systems level study is in understanding metabolic adaptations in different conditions such as *in vivo* environments for pathogens.

## Conclusion

In this study we gain comprehensive understanding of metabolic repertoire of Msm and its phenotypic response to different nutrient conditions. It can be inferred that many alternate nutrients are capable of being efficiently utilized by Msm as carbon and nitrogen sources when compared to Mtb and *M. bovis* strain. The comparative study for carbohydrate import systems of Mtb and Msm reveals larger number of genes involved in the mechanism and also expressed in reference condition [11]. This suggests the possibility of *Msm* to use alternate carbohydrates when present in the environment and also its relative faster growth when compared to the pathogenic counterparts. While the genes responsible for central metabolism are expressed in the reference medium, the expression of additional genes cannot be ruled out when provided with alternate nutrients. Thus, in the present study, experiments were performed to analyse the expression profile of the organism to infer the feasible metabolic pathways and also to derive the set of nutrients favourable for its growth. Integration of transcriptomic and phenotypic data along with functional annotation of the genome provides us insights into the biochemical repertoire of pathways possible when the medium is supplemented with an array of nutrients.

## Methods

### Functional annotation of Msm genome

Genome sequence for Msm was downloaded from TB Database (TBDB) [10]. The genome annotation as available for each locus was obtained from multiple sources, mainly TB Database [10], Smegmalist, Tuberculist [24] and xBASE [64]. Bidirectional BLAST searches [65] were performed to identify the homologous proteins present in Mtb. Functional categories were assigned to these homologues based upon Tuberculist classification where possible. In certain cases, more than 1 functional category was identified for some genes in Msm using the above method, so the most relevant functional category was assigned by manual curation. Pathway assignments for enzymes were initially obtained from an automated protocol from BioCyc [27]. The individual gene annotations were systematically compared to those from TBDB and verified for consistency. Additional pathway assignments were added as necessary.

### Transcriptome analysis

#### a) Strain and culture condition

*M. smegmatis* MC^2^ 155 wildtype culture was grown in Middlebrook 7H9 media until it reached 0.2-0.3 O.D_600_. Once the O.D was reached, 20 ml of the culture was pelleted down, and the supernatant was discarded. The pellet was resuspended in 100 μl of 1× PBS. The pellet was snap freezed in liquid nitrogen and stored in -80C until RNA extraction was carried out.

#### b) RNA extraction

RNA extraction was done using Qiagen’s RNeasy minikit (Cat#74104). The RNA quality was checked using Bioanalyzer. Labelling was done using Agilent’s Quick-Amp labeling Kit. Random hexamer method of labeling was done followed by T7 promoter based-linear amplification to generate labeled complementary RNA (One-Color Microarray-Based Gene Expression Analysis). Hybridization was performed using Agilent’s In situ Hybridzation kit 5188–5242. Chips used for microarray were customized for *M. smegmatis* MC^2^ 1558×15k Array AMADID: 020791 (Genotypic Technology, Bangalore, India).

#### c) Transcriptome data analysis

The raw data obtained from experiments have been normalized using GeneSpring GX 12.6.1 software. Intra-array normalization deals with variability within a single array. In intra-array normalization, gProcessed signal (dye normalized background subtracted signal intensity) is log transformed and then for each of the array elements, the 75^th^ percentile value is calculated separately. In each sample the log transformed intensity values for each probe is subtracted by the calculated 75^th^ percentile value of the respective array and expression values are obtained. Similarly 50^th^ and 25^th^ percentile normalization was calculated for the dataset.

#### d) Clustering

Hierarchical clustering of the normalized data was performed using GeneSpring GX 12.6.1 software. Pearson correlation coefficient to measure similarity between expression profiles and average linkage method was used for clustering genes.

### Metabolic network feasibility analysis

Analysis was carried out to map the gene expression data onto the metabolic network derived from KEGG [26] and Biocyc [27] for *M. smegmatis* MC^2^ 155. Based upon the expression profile for each locus in the individual pathway, we mapped the corresponding 25^th^, 50^th^ and 75^th^ percentile values to infer feasible metabolic pathways in the network.

### Phenome analysis

#### a) Bacterial strains, growth conditions and chemicals

*Mycobacterium smegmatis* MC^2^ 155 were cultivated at 37°C in Middlebrook 7H9 broth with 0.2% (vol/vol) glycerol and 0.1% (wt/vol) Tween 80 or on Middlebrook media 7H10 agar plates supplemented with 0.2% (vol/vol) glycerol and supplemented with OAD (oleic acid, albumin, dextrose).

#### b) PM measurements

Phenotype microarray experiments were carried out following standard Biolog Inc. (http://www.biolog.com/) protocols as provided by the supplier. To prepare the inoculum for Phenotypic microarray plates (PM01 for carbon source, PM03 for nitrogen source, PM05 for other nutrient supplement), bacteria colonies were grown in Middlebrook 7H10 medium containing 10% (v/v) albumin dextrose (AD) enrichment and 0.05% (v/v) Tween 80. Bacteria were harvested at 48 hrs. The *M. smegmatis* strains were re-suspended in inoculating fluid so as to have 81% transmittance. The Biolog plates PM03 and PM05 contain dextrose as a carbon source in the PM additive.

PM plates were inoculated with 100 μl of the mixture made up with the following volumes per plate: Middlebrook 7H9 broth at 1.2× (10 ml), Dye mix G at 100× (0.12 ml), PM additive appropriate to the plate at 12× (1 ml) and bacteria in the media at 13.64× (0.88 ml). For each plate, the final volume of mixture was 12 ml. After plate inoculation, the plates were transferred to an OmniLog (Biolog, Inc.) incubator and incubated at 37°C for 4 days and monitored for color change due to dye reduction in the wells. To have biological replicates, separate inocula were used in the experiment.

#### c) Data processing and analysis

Data were analysed initially with OmniLog-PM software for gathering the kinetic values or respiration rates. Then for further analysis, data aggregation, discretization and clustering of the biological replicates in each PM plate types were carried out using OPM package available in R. MATLAB R2011b Toolboxes were used for clustering and correlation analysis across PM plates.

## Additional files

Additional file 1:
**Functional categories for the** ***M. smegmatis*** **MC** ^**2**^ **155 genome.**
Additional file 2:
**The gene expression profile (25** ^**th**^ **,** **50** ^**th**^ **and 75** ^**th**^ **percentile) and pathway mapping.**
Additional file 3:
**Gene expression profile mapped onto representative pathways.** (a) Glycolysis-TCA-Glyoxylate shunt, (b) Serine biosynthesis and (c) Alanine biosynthesis. Additional file 4:
**Phenotypic microarray analysis.** The phenotypic microarray observation for PM1 for high growth nutrient conditions explored and pathway mapping along with the enzymes catalyzing the reaction (Sheet 1). Details for all the PM plate compositions and description about cluster is provided in Sheet 2. Additional file 5:
**The growth profile of Msm.** The profile is as observed in (a) reference condition (batch culture) and (b) reference well from PM plate. Additional file 6:
**XY and Level plot of PM plates** **(a)** **PM3 and** **(b)** **PM5.**
Additional file 7:
**Cluster analysis for PM plates** **(a)** **PM3 and** **(b)** **PM5.**
Additional file 8:
**KEGG metabolic map overlaid with compounds probed by PM analysis.** The red dots represent high growth, blue dots are moderate growth, while green dots represent low growth promoting compounds. Additional file 9:
**Gene expression profile mapped onto genes annotated as Transporters in KEGG.**
Additional file 10:
**Transporters and their expression profile.** A list of transporters annotated as part of functional category with their expression profile at 75^th^ percentile normalization.

## Abbreviations

Msm: Mycobacterium smegmatis MC^2^ 155
Mtb: Mycobacterium tuberculosis H37Rv
PM: Phenotypic microarray
BLAST: Basic Local Alignment Search Tool
Mbp: Mega basepair
EC: Enzyme Commission
TCA: Tricarboxylic acid
E.coli: *Escherichia coli*
