# Supplementary figures and images for: Identifying feasible metabolic routes in Mycobacterium smegmatis and possible alterations under diverse nutrient conditions
Source: BMC Microbiol. 2014 Nov 18;14:276. doi: 10.1186/s12866-014-0276-5 (PMC4248442; doi:10.1186/s12866-014-0276-5)

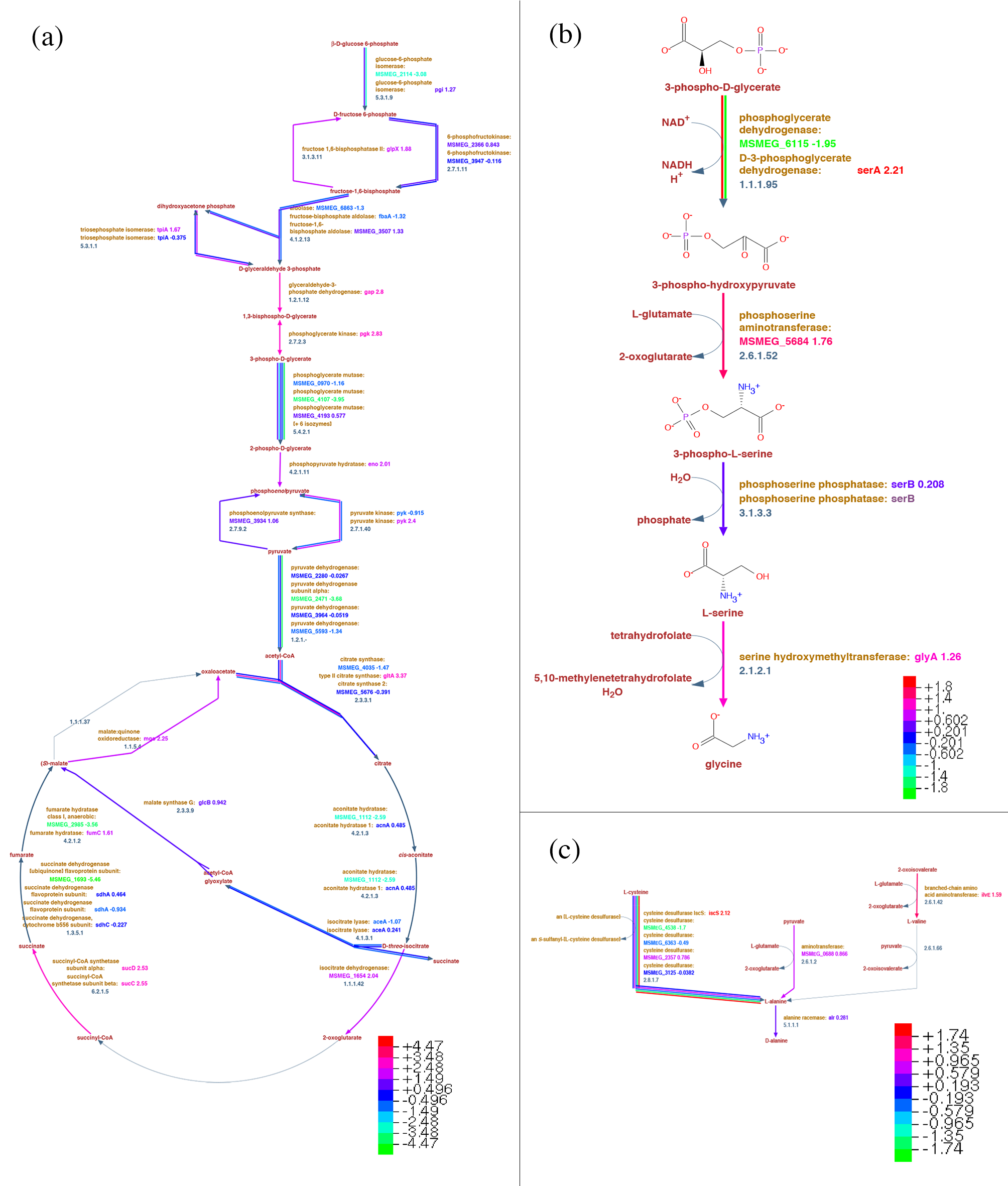

Supplement: Additional file 3: — Gene expression profile mapped onto representative pathways. (a) Glycolysis-TCA-Glyoxylate shunt, (b) Serine biosynthesis and (c) Alanine biosynthesis. [file 12866_2014_276_MOESM3_ESM.tiff]

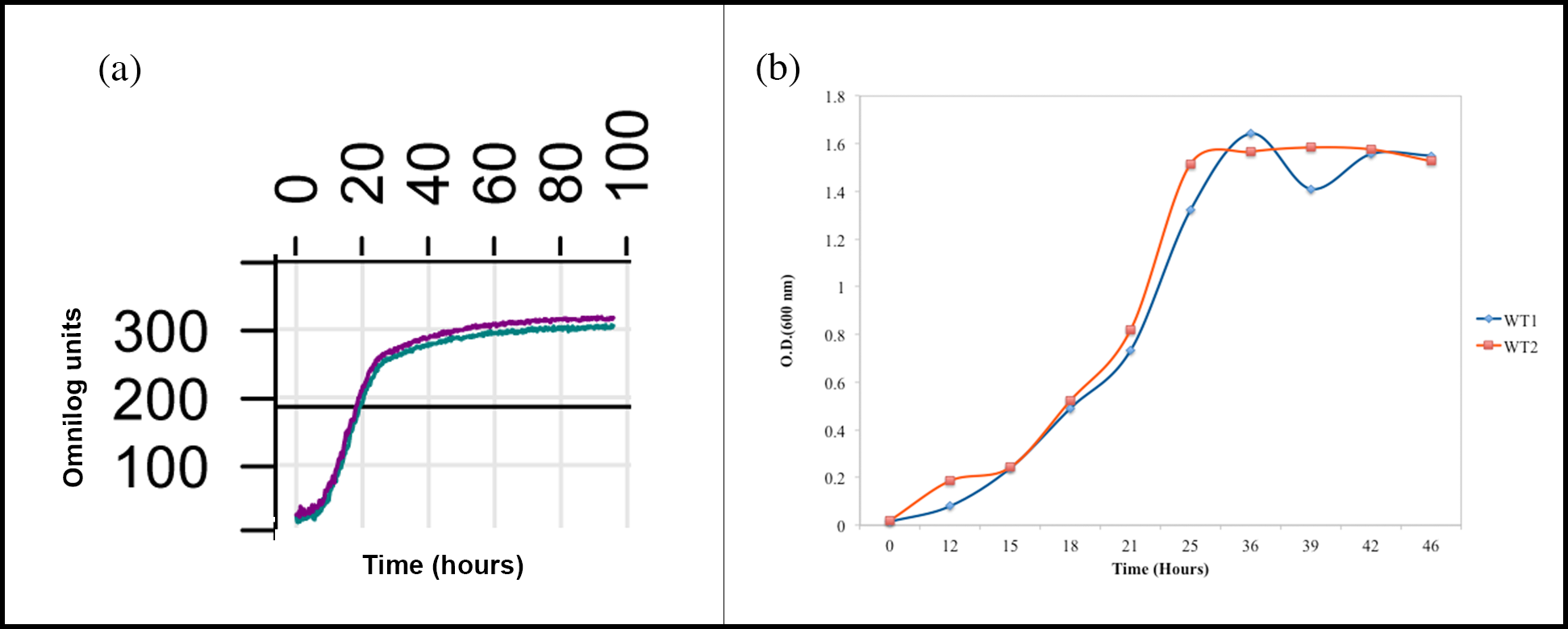

Supplement: Additional file 5: — The growth profile of Msm. The profile is as observed in (a) reference condition (batch culture) and (b) reference well from PM plate. [file 12866_2014_276_MOESM5_ESM.tiff]

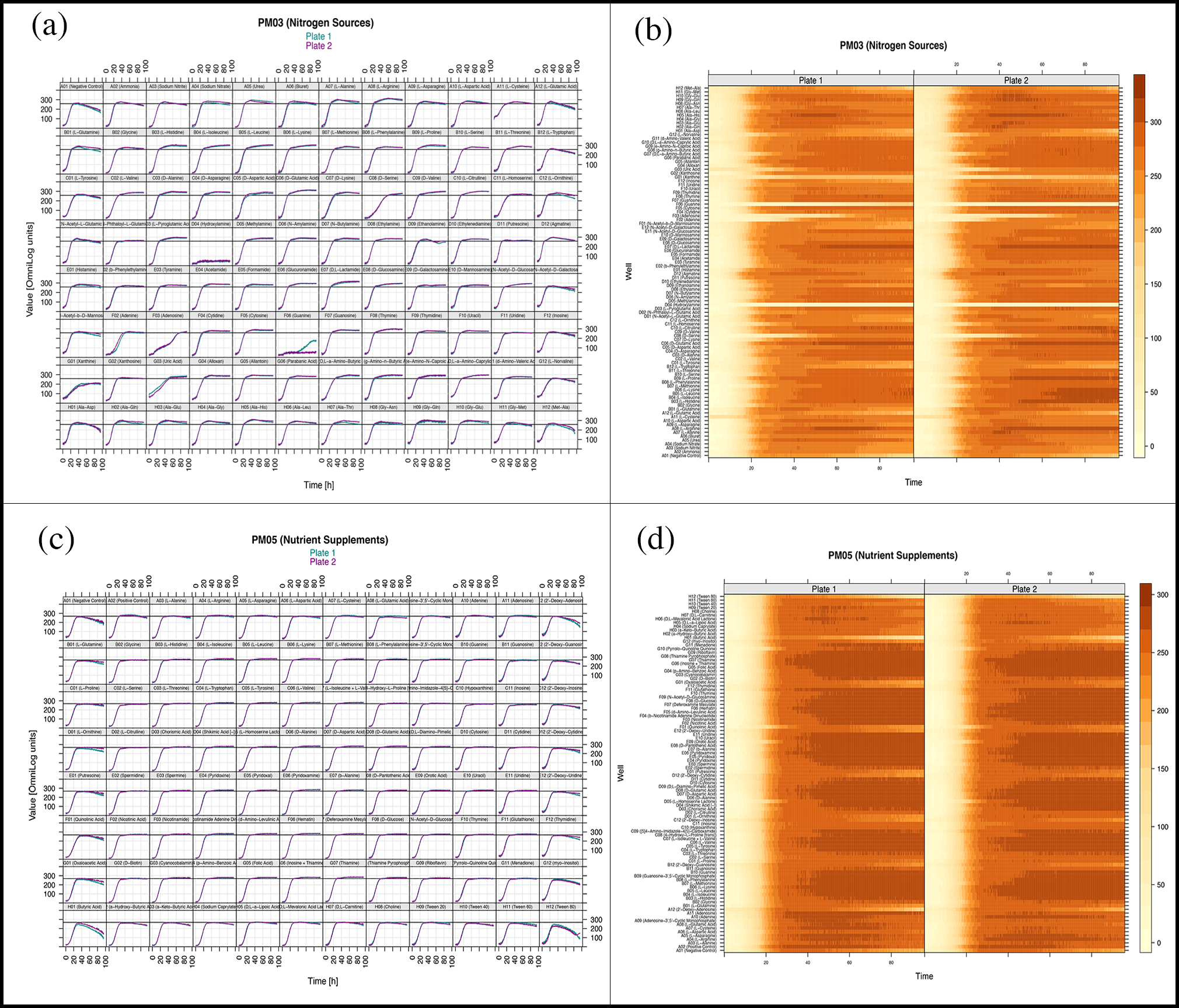

Supplement: Additional file 6: — XY and Level plot of PM plates (a) PM3 and (b) PM5. [file 12866_2014_276_MOESM6_ESM.tiff]

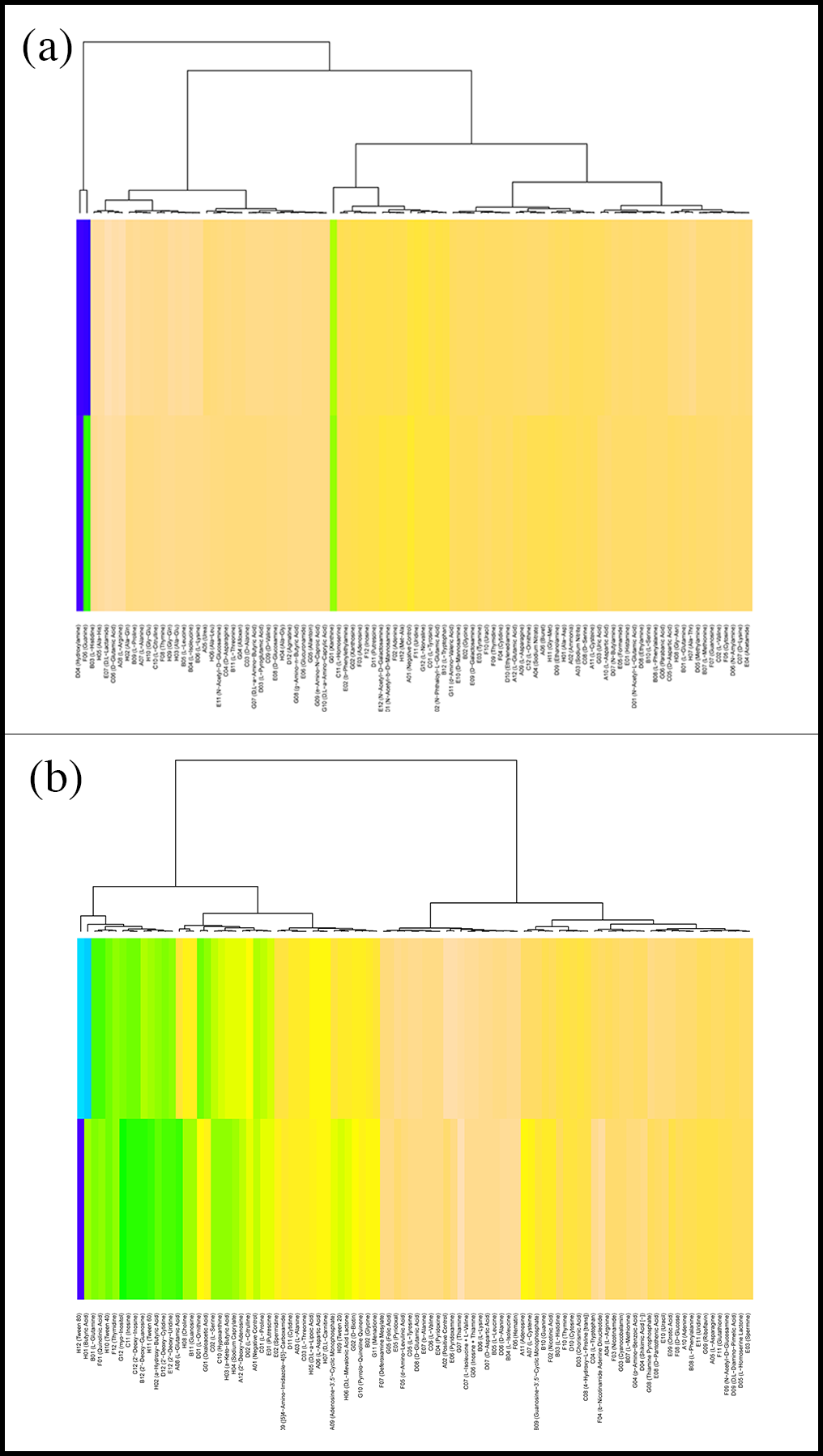

Supplement: Additional file 7: — Cluster analysis for PM plates (a) PM3 and (b) PM5. [file 12866_2014_276_MOESM7_ESM.tiff]

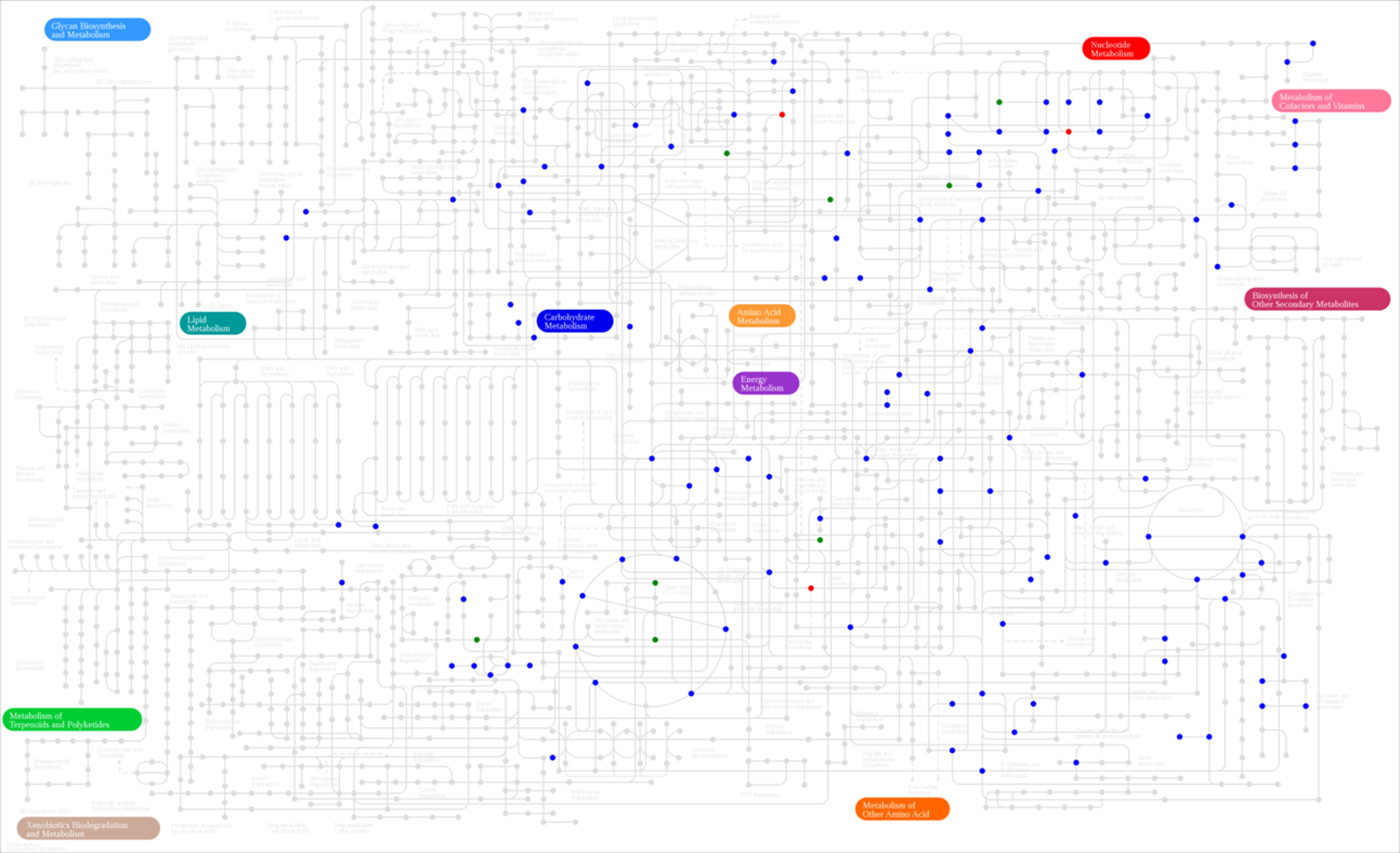

Supplement: Additional file 8: — KEGG metabolic map overlaid with compounds probed by PM analysis. The red dots represent high growth, blue dots are moderate growth, while green dots represent low growth promoting compounds. [file 12866_2014_276_MOESM8_ESM.tiff]

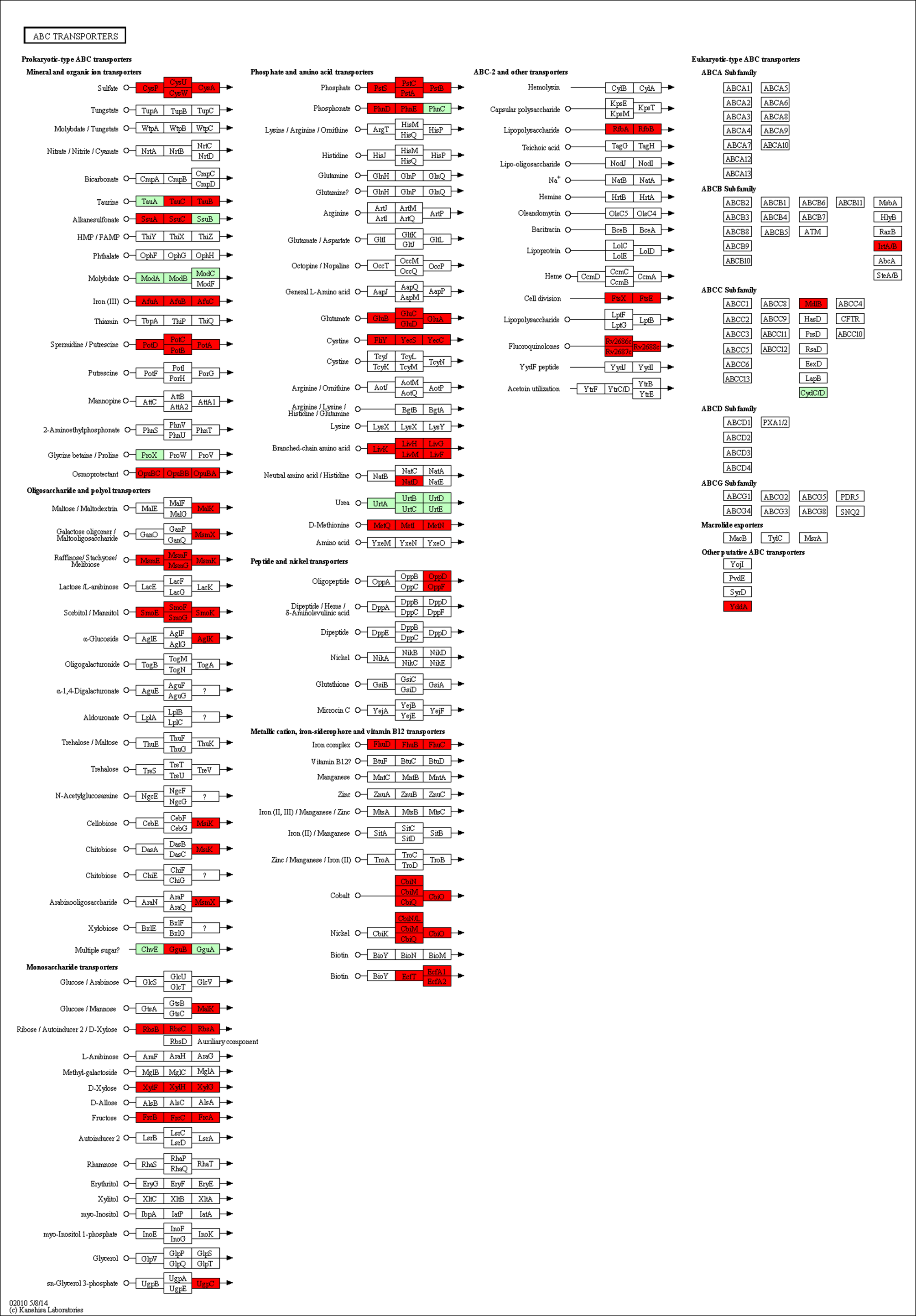

Supplement: Additional file 9: — Gene expression profile mapped onto genes annotated as Transporters in KEGG. [file 12866_2014_276_MOESM9_ESM.tiff]
